# Hepatitis E Virus Mediates Renal Injury via the Interaction between the Immune Cells and Renal Epithelium

**DOI:** 10.3390/vaccines8030454

**Published:** 2020-08-14

**Authors:** Mohamed A. El-Mokhtar, Mohamed Ismail Seddik, Asmaa Osman, Sara Adel, Essam M. Abdel Aziz, Sahar A. Mandour, Nasreldin Mohammed, Mohamed A. Zarzour, Lobna Abdel-Wahid, Eman Radwan, Ibrahim M. Sayed

**Affiliations:** 1 Department of Medical Microbiology and Immunology, Faculty of Medicine, Assiut University, Assiut 71515, Egypt; ma_mokhtar@yahoo.com; 2 Department of Clinical Pathology, Faculty of Medicine, Assiut University, Assiut 71515, Egypt; moh.ismail310@aun.edu.eg (M.I.S.); Asmaa.osama@must.edu.eg (A.O.); 3 Department of Clinical Pathology, Faculty of Medicine, Al-Azhar University, Assiut 71515, Egypt; SaraMohamed.44@azhar.edu.eg; 4 Department of Internal Medicine, Nephrology Division, Faculty of Medicine, Assiut University, Assiut 71515, Egypt; essam.nephro@gmail.com; 5 Department of Microbiology and Immunology, Faculty of Pharmacy, Deraya University, Minia 66111, Egypt; sahar.mandour@deraya.edu.eg; 6 Department of Urology and Renal Transplantation Centre, Faculty of Medicine, Assiut University Hospital, Assiut 71515, Egypt; nasreldin1975@yahoo.com (N.M.); Dr.mohamedali815@gmail.com (M.A.Z.); 7 Department of Internal Medicine, Faculty of Medicine, Assiut University, Assiut 71515, Egypt; lobna_wahid@yahoo.com; 8 Department of Medical Biochemistry, Faculty of Medicine, Assiut University, Assiut 71515, Egypt; emanradwan@aun.edu.eg; 9 Department of Pathology, School of Medicine, University of California, San Diego, La Jolla, CA 92093, USA

**Keywords:** HEV, renal disorder, immune-mediated, proximal tubular, inflammatory cytokines, chemokines, kidney injury, IFN-γ

## Abstract

Renal disorders are associated with Hepatitis E virus (HEV) infection. Progression to end-stage renal disease and acute kidney injury are complications associated with HEV infection. The mechanisms by which HEV mediates the glomerular diseases remain unclear. CD10^+^/CD13^+^ primary proximal tubular (PT) epithelial cells, isolated from healthy donors, were infected with HEV. Inflammatory markers and kidney injury markers were assessed in the presence or absence of peripheral blood mononuclear cells (PBMCs) isolated from the same donors. HEV replicated efficiently in the PT cells as shown by the increase in HEV load over time and the expression of capsid Ag. In the absence of PBMCs, HEV was not nephrotoxic, with no direct effect on the transcription of chemokines (Cxcl-9, Cxcl-10, and Cxcl-11) nor the kidney injury markers (kidney injury molecule 1 (KIM-1), neutrophil gelatinase-associated lipocalin (NGAL), and interleukin 18 (lL-18)). While higher inflammatory responses, upregulation of chemokines and kidney injury markers expression, and signs of nephrotoxicity were recorded in HEV-infected PT cells cocultured with PBMCs. Interestingly, a significantly higher level of IFN-γ was released in the PBMCs-PT coculture compared to PT alone during HEV infection. In conclusion: The crosstalk between immune cells and renal epithelium and the signal axes IFN-γ/chemokines and IL-18 could be the immune-mediated mechanisms of HEV-induced renal disorder.

## 1. Introduction

Hepatitis E virus (HEV) is a small icosahedral positive sense single-strand RNA virus that belongs to the Orthohepevirus genus of the Hepeviridae family [1]. The Orthohepevirus A includes eight genotypes (gts; HEV-1-HEV-8), at least five gts are infectious to humans [2,3]. HEV-1, HEV-2, HEV-3, HEV-4, and HEV-7 cause infection to humans, while other HEV isolates such as HEV-5, HEV-6, and HEV-8 are not confirmed as human pathogens [2,4]. There are four different transmission modes for HEV infection: fecal–oral, food-borne, blood-borne, and vertical transmission [4,5]. The fecal–oral route, transmitted by the drinking of contaminated water, is the most common mode of HEV transmission globally and is caused by HEV-1 and HEV-2 [3]. Food-borne HEV infection, caused by HEV-3, HEV-4, and HEV-7, is transmitted by the ingestion of raw or undercooked products from infected animals, such as pigs, wild boars, rabbits, deer, and camels [6,7,8]. Transfusion of contaminated whole blood or blood products is also associated with HEV infection [9]. Vertical or perinatal transmission from mother to child has been documented and it is associated with severe outcomes, especially with HEV-1 infection [10].

The HEV genome is about 7.2 kilobases long and it contains three open reading frames (ORF). ORF1, located at the 5’ end of the genome, encodes the enzymes required for HEV replication such as RNA dependent RNA polymerase, methyltransferase, helicase, and cysteine protease. ORF2, located at the 3’ end of the genome, encodes the immunogenic capsid protein and ORF3, overlaps with ORF1 and ORF2, encodes a small phosphoprotein that plays a role in the viral morphogenesis and egress from the infected cells [11].

HEV causes acute, chronic, and extrahepatic manifestations. Chronic HEV infections have developed in immunocompromised patients such as organ transplant patients, HIV infected patients, and patients with hematological disorders [12,13]. Several extrahepatic manifestations have been reported in association with HEV infection such as neurological disorders, renal disorders, cryoglobulinemia, acute pancreatitis, and hematological disorders [14]. Renal disorders associated with HEV infection include membranous nephropathy (MN), IgA nephropathy, membranoproliferative glomerulonephritis (MPGN), and acute kidney injury (AKI) [15,16,17]. Extrahepatic replication of HEV in the kidney was also documented using in vivo animal models such as nonhuman primates, swine, and rabbits [18,19,20]. Moreover, HEV was detected in the urine of HEV-infected patients and in vivo animal models [20,21].

The mechanisms by which HEV can induce renal disorders are not completely understood. HEV could cause glomerular disorders through the immune-mediated mechanisms in an analogous way to immune-mediated renal disorders caused by other hepatotropic viruses such as HBV and HCV. HCV infection is associated with several glomerulopathies such as MPGN, MN, IgA nephropathy, and interstitial nephritis. The pathogenesis of HCV induced glomerulopathies is mostly through immune-mediated mechanisms [22]. Similarly, HBV caused glomerulonephritis with acute thrombotic microangiopathy in a kidney transplant recipient though immune complexes mechanisms [23]. However, the direct effect of HEV on the kidney cannot be ruled out.

Virus infection has been found as an important causative agent of acute kidney injury (AKI) such as infections associated with dengue virus and zika virus [24,25]. The most common biomarkers of AKI, including neutrophil gelatinase-associated lipocalin (NGAL), kidney injury molecule 1 (KIM-1), and interleukin 18 (lL-18) [26]. Progression to end-stage renal disease, AKI, and/or acute on chronic kidney diseases (CKD) was also associated with HEV infection [15,16,17,27,28].

Up to our knowledge, this is the first report that describes the possible mechanisms of HEV mediated renal disorders. Herein, we isolated the PT epithelial cells from healthy donors, infected them in vitro with HEV inoculum, and then we assessed the expression of inflammatory and kidney injury markers in the presence or absence of immune cells (PBMCs) isolated from the same donors. We found that HEV replicated efficiently in PT cells, and slightly elevated the inflammatory response, without direct nephrotoxicity. Coculture of PBMCs with HEV-infected PT cells exacerbated the inflammatory response and induced kidney injury.

## 2. Materials and Methods

### 2.1. Subjects

Kidney biopsies were obtained from patients admitted to Assiut University Hospitals for screening renal or urinary tract cancers. Blood samples were collected from the same donors and passed through Ficoll–Hypaque density gradient centrifugation for isolation of peripheral blood mononuclear cells (PBMCs) from the buffy coat. All donors were tested negative for viral hepatitis markers (HAV, HBV, HCV, and HEV markers) at the time of sample collection according to the protocol of Assiut University Hospitals as described previously [29,30,31]. All participating subjects provided written informed consent. The study design was approved by the Institutional Review Board at the Faculty of Medicine, Assiut University, Egypt following the provisions of the Declaration of Helsinki (IRB no.17300400)

### 2.2. Isolation and Characterization of Double-Positive Proximal Tubular (PT) Epithelial Cells

The isolation of proximal tubular (PT) cells was performed as described previously with slight modification in the procedure [32]. Briefly, renal cortical tissues were digested using collagenase IV (enzymatic activity: 200 U/mL; Invitrogen) for 30 min at 37 °C. The cell suspension was filtered, washed, and centrifuged. The cell pellets were dissolved in DMEM-F12 media (Gibco, Thermo Fisher Scientific, Grand Island, NY, USA) medium containing specific cocktails: 2% FBS (Gibco, Thermo Fisher Scientific, Grand Island, NY, USA), 10 ng/mL EGF, 1% penicillin/streptomycin, 1% L-glutamine, 15 mM HEPES, 50 mM hydrocortisone, 5 µg/mL insulin, 5 µg/mL transferrin, and 50 nM sodium selenite (Sigma-Aldrich Chemie Gmbh, Munich, Germany), incubated at 37 °C under 5% CO_2_ in a humidified atmosphere. The isolation of CD10^+^/CD13^+^ PT cells was done using microbeads. To check the purity of the isolated PT cells, the cells were double-labeled with phycoerythrin (PE)-conjugated anti-CD13 or with allophycocyanin (APC)-conjugated anti-CD10 (eBioscience, San Diego, CA, USA). Isotype matched antibodies were used as staining controls. Cells were acquired using the FACSCalibur flow cytometer (BD Biosciences, San Jose, CA, USA) and the data were analyzed using FlowJo software 7.6.1 (Tree Star Inc., Ashland, OR, USA). Also the markers of CD10^+^/CD13^+^ PT cells were measured by qPCR. PT cells were used at passages 2–6 in the infection experiments before they showed signs of senescence.

### 2.3. Measurements of Trans-Epithelial Electrical Resistance (TEER)

Cells were seeded onto a transwell chamber (Corning) without matrix, at a density of 1 × 10^5^ cells per well. At confluence, the TEER was measured using a Millicell^®^ ERS-2 Voltohmmeter (Millipore, Burlington, MA, USA). TEER readings are expressed in Ω.cm^2^.

### 2.4. Infection of CD10^+^/CD13^+^ PT Epithelial Cells with HEV Preparation

The infection of CD10^+^/CD13^+^ PT epithelial cells with HEV-1 was done as described previously with slight modification [29,33]. Briefly, one day before the infection, 1 × 10^5^ cells/well of PT epithelial cells were seeded in 0.4 µ transwells and placed in a 24-well plate. A filtered 10% *w/v* fecal preparation was added to the basolateral side of the cells at a dose of 10^6^ IU/well and incubated for 6 h. After that, the inoculum was removed and replaced with fresh culture medium. Supernatants were collected at 0, 2, 4, 6, 8, and 10 days postinfection, stored −80 °C till processing. Quantification of HEV RNA was done as described previously [34,35,36,37], and the detection of intracellular and extracellular HEV capsid protein was assessed as before [29]. As a control experiment, PT cells were challenged with UV-inactivated HEV-1. Additionally, PBMCS were challenged with UV-inactivated HEV-1 as described previously [33].

### 2.5. Assessment of HEV Viral Load by qPCR

Quantification of HEV RNA was done as described previously [34,35,36,37]. Briefly, viral RNA was extracted from the supernatants and cell lysates collected from PT epithelial cells challenged or not with HEV using QIAamp Viral RNA Mini Kit (Qiagen, Hilden, Germany) and an RNeasy Mini Kit (Qiagen, Hilden, Germany) according to the manufacturer’s instructions. HEV RNA was quantified using primers targeting HEV ORF2/3 as described before [34,35,36]. The limit of quantification (LOQ) of our assay is 300 IU/mL for undiluted samples.

### 2.6. Detection of Intracellular and Extracellular HEV Capsid Protein

Detection of HEV ORF2 Ag in the infected cells was done by flow cytometry as described previously [29,33]. CD10^+^/CD13^+^ PT cells were challenged with the HEV-1, the cells were fixed at Day 10 postinfection for the detection of intracellular HEV ORF2 Ag. Detection of extracellular HEV capsid protein in cell culture supernatants was performed using the HEV Ag ELISA^Plus^ assay (Beijing Wantai Biological Pharmaceutical Co., Beijing, China) according to the manufacturer’s instructions, with slight modifications in the procedure of cut-off (C.O.) calculation as described previously [29,33].

### 2.7. CoCulture of CD10^+^/CD13^+^ PT Epithelial Cells with PBMCs

For coculture experiments, CD10^+^/CD13^+^ PT epithelial cells were challenged with HEV for 7 days as described earlier and then PBMCs were added and incubated with the cells and HEV for an additional 3 days. Lysis of cells and collection of the supernatant were done on Day 10 for further processing. As a control group, PT/PBMCs coculture were challenged with Ultraviolet (UV)-inactivated HEV inoculum as described previously [33].

### 2.8. Test the Effect of HEV Infection on the Transcriptome of CD10/CD13 PT Epithelial Cells

Total cellular RNA was extracted from CD10^+^/CD13^+^ PT using the RNeasy Mini Kit (Qiagen, Hilden, Germany) according to the manufacturer’s instructions. RNA was converted into complementary DNA (cDNA) using MultiScribe reverse transcriptase according to the manufacturer’s instructions (Invitrogen, Waltham, MA, USA). Quantitative real-time polymerase chain reaction (qPCR) was carried out using SYBR green master mix (Applied Biosystems, Foster, CA, USA) on 7500 Fast Real-Time PCR (Applied Biosystems, Foster, CA, USA) for target genes and normalized to the housekeeping gene (18s rRNA) using the 2^−ΔΔCt^ method. The sequences of primers used in this study are listed in Table S1 in the supplementary material and methods.

### 2.9. LDH Assay

Supernatants were collected from uninfected PT cells, uninfected PT cells cocultured with PBMCs, HEV-infected PT, and HEV-infected PT cocultured with PBMCs were assayed for LDH using LDHGlo^TM^ Cytotoxicity Assay kit (Promega, Madison, WI, USA) according to the manufacturer’s instruction as described previously [38]. Briefly, supernatants were diluted 1/100 in LDH storage buffer, and then, an equal volume of LDH Detection Reagent was added to the diluted sample. The LDH activity was determined by measurement of luminescence. LDH-positive control (purified Lactate Dehydrogenase from rabbit muscle) was included in each assay. LDH-positive control and standard curves were included and generated according to the manufacturer’s instructions. Culture media was used to determine the medium background.

### 2.10. Measurement the Level of Inflammatory Cytokines Released after HEV Infection

Supernatants were collected from uninfected and HEV-infected PT cells cocultured or not with PBMCs at Day 10 pi were tested for IFN-γ by ELISA kits (R&D Systems, Minneapolis, MN, USA) according to the manufacturer’s instructions.

### 2.11. Treatment of PT Cells with IFN-γ 

PT cells were seeded as described in the previous section, then they were treated with IFN-γ (Sigma-Aldrich, Munch, Germany) at the concentration of 1 ng/mL for 3 days.

### 2.12. Statistics

Statistical analyses were performed using the GraphPad Prism software 6 (GraphPad Software, La Jolla, CA, USA) using the unpaired student’s *t*-test. *p* < 0.05 was considered significant.

## 3. Results

### 3.1. Isolation and Characterization of Primary Human PT Epithelial Cells

**Figure 1:**
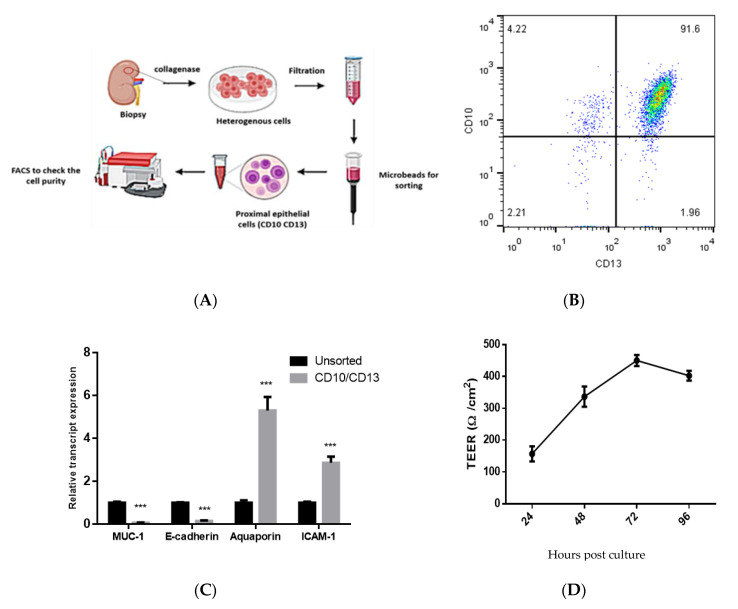
Isolation and characterization of primary human proximal tubular (PT) epithelial cells. (**A**) Schematic flow showing the isolation of PT epithelial cells from a kidney biopsy, and sorting of the cells using microbeads to isolate CD10^+^/CD13^+^ cells. (**B**) The isolated human PT cells were analyzed by flow cytometry to check for purity and the isolated cells were stained with anti-CD10 and anti-CD13. (**C**) The isolated human PT cells were checked for MUC-1, E-cadherin, Aquaporin1, and ICAM-1 mRNA expression by qPCR. Black columns represent unsorted cells, and grey columns represent CD10^+^/CD13^+^ cells. Depicted are the mean values of three independent experiments ± SEM. *** indicates *p* < 0.001 as determined by student’s *t*-test. (**D**) Transepithelial electrical resistance (TEER) was measured in the cultured CD10^+^/CD13^+^ PT cells over time.

Renal cortical tissues were collected from fresh nephrectomy specimens of patients admitted to Assiut University Hospitals to screen for renal or urinary tract cancer. The pathological examination revealed that the samples were not cancerous and did not include significant parenchymal lesions. Double-positive CD10^+^/CD13^+^ PT cells were sorted from the heterogeneous cell mixtures by positive selection (Figure 1A). To assess the purity of the isolated cells, we checked the CD10 (neutral endopeptidase) and CD13 (aminopeptidase M) markers by flow cytometry and we assessed the expression of specific cell markers by qPCR. Over 85% of the isolated cells were CD10^+^/CD13^+^ (Figure 1B), and they had high expression of intercellular adhesion molecule 1 (ICAM-1) and aquaporin 1 (specific markers for the differentiated PT), and reduced expression of transmembrane mucin 1 (MUC-1) and E-cadherin (specific distal tubule and collecting duct marker) compared to the unsorted cell population (Figure 1C). Besides, the cells had a characteristic epithelial structure, and the TEER value was increased over time suggesting the formation of a tight junction between the polarized epithelial cells (Figure 1D). The PT cells were phenotypically stable; i.e., expressed CD10 and CD13 over six passages on cultures before they showed signs of senescence.

### 3.2. Infection of the CD10^+^/CD13^+^ PT Epithelial Cells with HEV Inoculum

**Figure 2:**
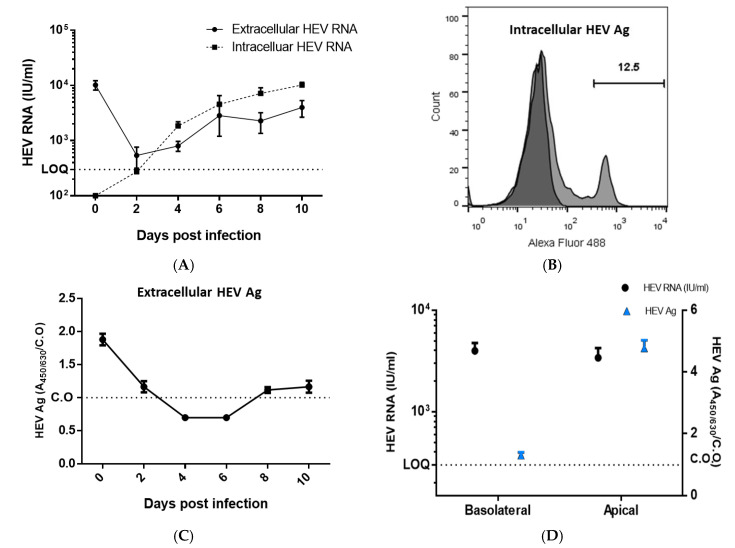
Infection of the CD10^+^/CD13^+^ PT epithelial cells with HEV inoculum. (**A**) Infection of polarized CD10^+^/CD13^+^ PT cells, cultured on transwell, with HEV-1 inoculum at the basolateral side. Intracellular (dotted line) and extracellular (solid line) HEV RNA was quantified by qPCR. LOQ: limit of quantification. Depicted are the mean values of three independent experiments ± SEM. (**B**) Representative gating strategy showing the expression of HEV ORF2 Ag in the PT cells infected with HEV. The dark histogram represents cells stained with the secondary A488 conjugated anti-mouse antibodies alone; the light shaded histogram represents cells stained by mouse anti-HEV-ORF2 followed by A488 conjugated anti-mouse antibody. (**C**) Supernatants collected from HEV-1 infected PT cells were tested for HEV ORF2 Ag by ELISA, C.O is the cutoff. Depicted are the mean values of three independent experiments ± SEM. (**D**) HEV RNA (black), and HEV Ag (blue) were measured at the basolateral and apical sides. Depicted are the mean values of three independent experiments ± SEM.

The PT cells were cultured on a transwell, and the polarized epithelial cells were infected with HEV-1 inoculum at the basolateral side. HEV RNA started to be increased in Day 2 (intracellular) and Day 4 (extracellular) postinfection (pi) and the viral load increased over time, reaching 1.1 × 10^4^ and 5.4 × 10^3^ at Day 10 pi for intracellular and extracellular HEV RNA, respectively (Figure 2A). Intracellular HEV capsid protein was detected in the infected PT cells at Day 10 pi by flow cytometry, and the percentage of HEV-ORF-2 positive cells was 13–20% (Figure 2B). The HEV ORF2 Ag was also accumulated extracellularly in the supernatants of HEV-infected cells over time; the level of HEV Ag was increased in both compartments (i.e., basal and apical side; Figure 2C). The HEV viral load was comparable in the basolateral and apical sides, while the level of HEV Ag was higher in the apical side compared to the basolateral side (Figure 2D).

### 3.3. HEV Infection Alone Slightly Elevated the Inflammatory Response with No Effect on the Transcription of Chemokines nor Kidney Injury Markers

**Figure 3:**
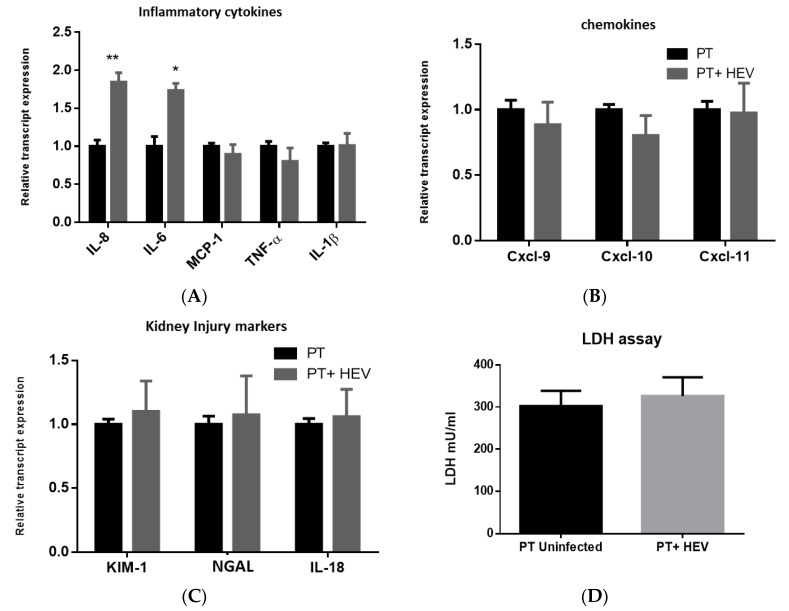
HEV infection alone did not affect the transcription of chemokines nor kidney injury markers of the PT epithelium. CD10+/CD13+ PT cells were challenged with HEV-1 for 10 days and then total cellular RNA was extracted and the mRNA expression level of proinflammatory markers (IL-8, IL-6, MCP-1, TNF-α, and IL-1β) (**A**), chemokines (Cxcl-9, Cxcl-10, and Cxcl-11) (**B**), and kidney injury transcripts (kidney injury molecule 1 (KIM-1), neutrophil gelatinase-associated lipocalin (NGAL), and interleukin 18 (lL-18)) (**C**) were assessed. The relative gene expression was determined by comparing the expression levels of these transcripts with mock cells. Black columns represent uninfected cells, and grey columns represent HEV infected cells. Data represent the mean +/− SEM of four separate experiments. *, **, indicates *p* ≤ 0.05 and *p* ≤ 0.01 as assayed by two-tailed Student’s *t*-test. (**D**) The level of LDH was measured in the supernatants of PT infected or not with HEV at Day 10 postinfection. Data represent the mean ± SEM of four separate experiments. Black columns represent uninfected cells, and grey columns represent HEV infected cells.

We assessed the effect of HEV infection of the transcriptome of PT cells for selected inflammatory cytokines, chemokines, and kidney injury markers. Regarding the inflammatory markers, we found that HEV infection slightly upregulated IL-8 and IL-6 (fold change around 1.8 to 2 compared to uninfected cells; Figure 3A), but it did not affect the expression of other inflammatory transcripts such as monocyte chemoattractant protein-1 (MCP-1), tumor necrosis factor-alpha (TNF-α), and Interleukin 1 beta (IL-1β) (Figure 3A). The expression of type I IFN (IFN-α and IFN-β) was not altered with HEV infection. While HEV infection did not alter the transcription of chemokines such as Cxcl-9, Cxcl-10, and Cxcl-11 nor the kidney injury markers such as KIM-1, NGAL, and IL-18 (Figure 3B,C). There was no significant difference in the LDH level between the supernatant collected from HEV-infected and noninfected PT cells (Figure 3D).

### 3.4. The CoCulture of PBMCs with the HEV-Infected PT Epithelial Cells Increased the Expression of Inflammatory Cytokines, Chemokines, and Kidney Injury Markers Produced from the Epithelium

**Figure 4:**
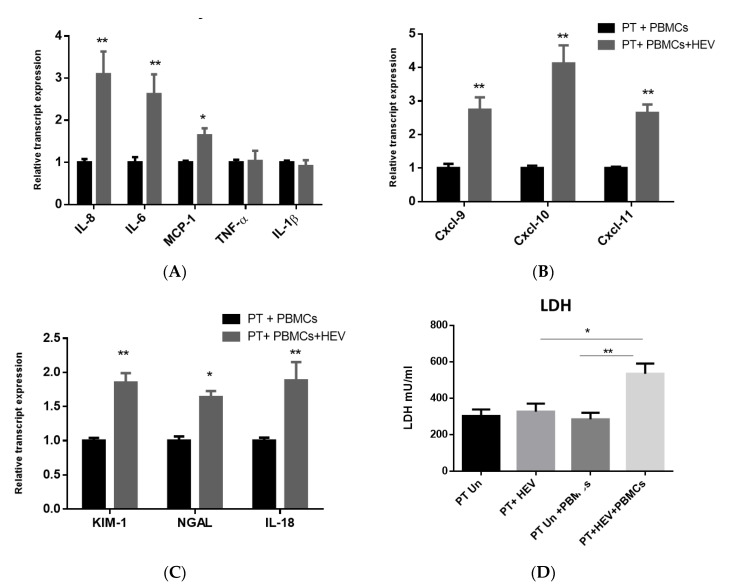
Coculture of PBMCs with the PT epithelial cells increased the inflammatory and kidney injury markers produced from the epithelium upon HEV infection. CD10^+^/CD13^+^ PT cells were infected with HEV-1 for 7 days and then PBMCs from the same donors were added for an additional 3 days. Total cellular RNA was extracted from the PT cells and the mRNA expression level of proinflammatory markers (IL-8, IL-6, MCP-1, TNF-α, and IL-1β) (**A**), chemokines (Cxcl-9, Cxcl-10, and Cxcl-11) (**B**), and kidney injury transcripts (KIM-1, NGAL, and IL-18) (**C**) were assessed. The relative gene expression was determined by comparing the expression levels of these transcripts with mock cells (PT + PBMCs). Black columns represent uninfected cocultured PT/PBMCs cells, and grey columns represent HEV infected PT/PBMCs cocultured cells. Data represent the mean ± SEM of four separate experiments. *, **, indicates *p* ≤ 0.05 and *p* ≤ 0.01 as assayed by two-tailed Student’s *t*-test. (**D**) LDH assay was performed on PT cells either uninfected or HEV-infected in the presence or absence of coculture with PBMCs.

Then, we asked if PBMCs can affect the inflammatory response and kidney injury markers released from the PT epithelial cells upon HEV infection. To assess this point, we cocultured the PT epithelial cells challenged or not with HEV, with PBMCs collected from the same donors from whom the PT epithelial cells were isolated. Control samples, in which the coculture of PT and PBMCs was not challenged with HEV, were included. First, we analyzed the effect of PBMCs on the expression of the inflammatory and kidney injury markers released from the PT cells in the absence of HEV infection. We did not notice a significant difference in the transcription of these markers. Compared to uninfected PT epithelial cells/PBMCs coculture, we found that the inflammatory cytokines such as IL-8, IL-6, and MCP-1 were significantly upregulated in the PT/PBMCs cocultured cells challenged with HEV (Figure 4A). Additionally, we detected an upregulation in the transcription of chemokines such as Cxcl-9, Cxcl-10, and Cxcl-11 and kidney injury markers such as KIM-1, NGAL, and IL-18 markers suggesting the start of kidney injury (Figure 4B,C). PT and/or PBMCS challenged with UV-inactivated HEV-1 did not show signs of active HEV replication (Figure S1). PT epithelial cells/PBMCs coculture were challenged with UV-inactivated HEV-1, and we did not record a significant change in the transcripts of the inflammatory cytokines, chemokines nor AKI markers (Figure S2). The level of LDH was significantly higher in HEV-infected PT cells cocultured with PBMCs compared to HEV-infected PT cells without PBMCs (Figure 4D).

### 3.5. Exacerbation of the Inflammatory Response Was Dependable on IFN-γ Produced from the PBMCs

**Figure 5:**
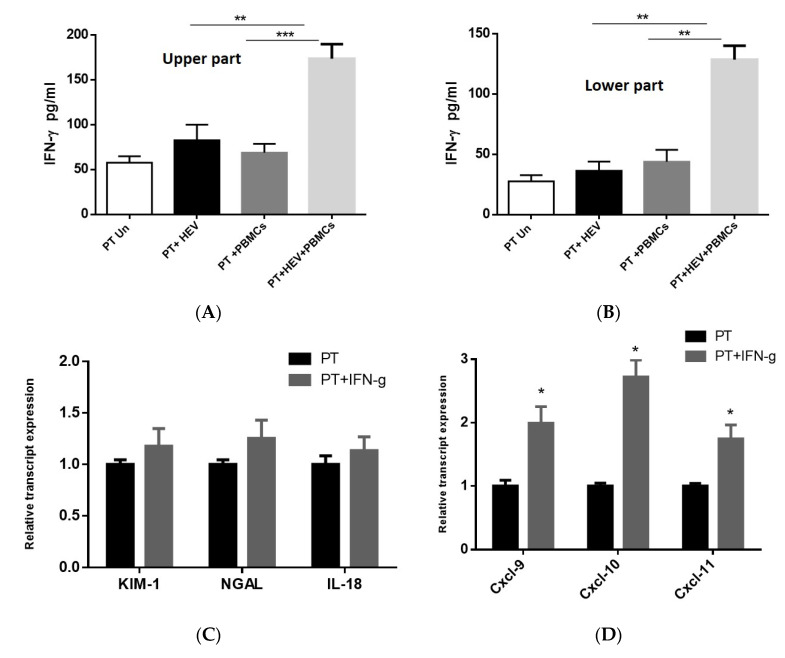
Exacerbation of the inflammatory response was dependable on IFN-γ produced from the PBMCs. PT cells, either uninfected or HEV-infected in the presence or absence of coculture with PBMCs, were assessed for IFN-γ. The level of IFN-γ was measured in the apical (upper compartment) (**A**) and basolateral (lower compartment) (**B**) of PT cells, PT cells cocultured with PBMCs challenged or not with HEV. PT cells were treated or not with IFN-γ (1 ng/mL) for 3 days. The relative expression levels of kidney injury markers (KIM-1, NGAL, and IL-18) (**C**) and chemokines (**D**) was compared in PT cells treated (gray) or not (black) with IFN-γ. Data represent the mean ± SEM of four separate experiments. *, **, and *** indicates *p* ≤ 0.05, *p* ≤ 0.01, and *p* ≤ 0.001 as assayed by two-tailed Student’s *t*-test.

Since the release of the chemokines from the PT cells and the degree of kidney injury are IFN-γ dependable [39,40,41], we assessed the level of IFN-γ produced from PT cells infected with HEV in the presence and absence of PBMCs. There was no significant difference in the level of IFN-γ produced from PT cells alone or PT cocultured with PBMCs in the absence of HEV infection. Additionally, the infection of the PT cells with HEV did not result in a significant release of IFN-γ in absence of PBMCs. In contrast, the coculture of PBMCs with HEV-infected PT cells led to a significant induction of IFN-γ in both apical and basolateral compartment (Figure 5A,B). To assess the direct effect of IFN-γ on the kidney injury, and chemokines stimulation, we treated PT cells with IFN-γ (1 ng/mL) for three days and then we assessed the effect of IFN-γ on the transcript level. Compared to PT cells alone, we found that IFN-γ upregulated the chemokines transcript (Cxcl-9, Cxcl-10, Cxcl-11) without effect on the transcript levels of kidney injury markers (KIM-1, NGAL, and IL-18; Figure 5C,D).

## 4. Discussion

Extrahepatic manifestations such as neurological disorders, renal disorders, cryoglobulinemia, acute pancreatitis, and hematological disorders have been reported in association with HEV infection [14]. Renal disorders reported during HEV infection include MPGN, IgA nephropathy, MN, nephroangiosclerosis, cryoglobulinemia associated glomerulonephritis, cholemic nephrosis, and acute renal failure [15,16,17]. The likelihood of a causal relationship between HEV infection and renal disorders is strong. Kamar et al. reported a strong association between HEV infection and renal impairment. They found that the glomerular filtration rate was impaired in kidney and liver transplant recipients during the acute and chronic phases of HEV infection. Out of 51 organ transplant recipients and HEV-infected patients, eight (15.6%) developed renal dysfunction with no other possible causes [16]. Not only immunocompromised patients but also HEV mediated renal disorders were reported in immunocompetent patients [42]. Another link between HEV infection and kidney is the excretion of HEV particles in the urine of acute and chronic HEV-infected patients [20]. Additionally, HEV was detected in the urine of in vivo animal models after challenging with HEV and developing chronicity [18,19,20]. Importantly, infection of naïve monkeys with the urine of HEV-infected monkeys led to the development of HEV infection, confirming that the released particles in the urine were infectious [20]. Although, most of the reported cases of HEV associated renal disorders are linked to HEV-3, renal disorders linked to other HEV genotypes such as HEV-1 and HEV-4 were also documented [17,43,44].

The underlying mechanism by which HEV infection may induce glomerular disease remains unclear. In general, the extrahepatic manifestations of HEV are mediated either by direct cytopathic effect due to the virus replication, or indirectly via various immune-mediated mechanisms [14]. The immune-mediated mechanisms of HEV-induced glomerular diseases are probably similar to HCV and/or HBV induced glomerular diseases which involve the excessive immune response against the viral antigens, deposition of the immune complexes, and the infiltration of the inflammatory cells around the deposits [14,45]. The direct effect of HEV on the kidney is not completely understood. Verschuuren et al. reported a case with fulminant HEV who developed a nonoliguric acute renal failure, and acute tubular necrosis probably due to the direct effect of HEV, since the renal biopsy of this patient showed no vascular abnormalities, and immune-fluorescence for IgA, IgG, IgM, heavy chains, k and l chains, and complement C3 was negative, suggesting that the renal impairment was not immune-mediated [46].

In this study, we isolated CD10^+^/CD13^+^ PT epithelial cells and cultivated them in vitro for HEV infection. We preferred to use the CD10^+^/CD13^+^ PT cells since these cells constitute a pure, functional and stable PT epithelial cell population, and they retain the epithelial characteristics over the long term, compared to other proximal cells that are mono-positive (CD10 or CD13 positive alone) or double negative cells which appear to be heterogeneous [32]. The purity of sorted cells was checked by flow cytometry (double labeling for CD10/CD13), and for MUC-1, E-cadherin, Aquaporin1, and ICAM-1 mRNA expression by qPCR. We found that over 85% of the cells were DP for CD10^+^/CD13^+^, and the transcript level of MUC-1 and E-cadherin, which are, the marker for distal and collecting tubules, was decreased, while the transcript level of Aquaporin1 and ICAM-1 were increased in the sorted cells compared to heterogeneous cells confirming that the sorted cells were PT epithelial cells [32,47].

In this study, we challenged the CD10^+^/CD13^+^ PT cells with HEV inoculum. HEV replicated efficiently in these cells as shown by the increase in HEV load over time and the expression of intracellular and extracellular capsid Ag, suggesting that the kidney is a target for the extrahepatic HEV replication. Similarly, the replication of HEV in the kidney was documented using in vivo animal models such as monkeys, rabbits, and swine; HEV negative-strand RNA and HEV Ag were detected in the kidney of these infected animals [18,19,20,48]. Besides, HEV RNA and HEV Ag were detected in the urine of HEV-infected patients, monkeys, and rabbits [18,19,20]. On the other hand, the replication of HEV-1 was inhibited in vitro in a swine kidney cell line, probably HEV-1 produced insufficient capsid protein for optimal assembly in swine cells [49]. Additionally, HEV was not replicating in the kidney of human liver chimeric mice, which was murine in its origin [36,50]

In this study, we found that HEV RNA load was comparable in both basolateral (direction of the bloodstream) and apical compartment (direction of the urine), while the level of HEV Ag as measured by A_450/630/_C.O. was higher in the apical side than the basolateral side. Consequently, the level of HEV Ag/HEV RNA was higher in the apical side compared to the basolateral side. Similarly, the urinary HEV Ag to RNA ratios were significantly higher than those for the blood in HEV-infected patients [20,21].

In this study, we assessed if HEV mediated kidney injury was due to the direct effect of HEV replication or through immune-mediated mechanisms by analyzing the inflammatory responses and kidney injury markers produced from the PT cells cocultured or not with PBMCs. In the absence of PBMCs, HEV infection slightly upregulated the inflammatory markers such as IL-8 and IL-6 but MCP-1, TNF-α, and IL-1β were not affected. The transcription of chemokines and the kidney injury markers were not affected by HEV infection. Similarly, the LDH level, a marker of cell cytotoxicity, was not changed upon HEV infection. On the other hand, higher inflammatory responses, upregulation of chemokines and kidney injury markers expression, and an increase in the level of LDH were recorded in HEV-infected PT cells cocultured with PBMCs. Collectively, these findings suggest that HEV infection alone is not nephrotoxic, and the renal manifestations associated with HEV infection are primarily due to immune-mediated mechanisms. In a parallel line, HEV is not hepatotoxic nor neurotoxic and the manifestations associated with HEV in these organs are immune-mediated mechanisms [35,51,52,53].

In this study, we measured the level of IFN-γ released in the coculture model of PT cell, HEV, and PBMCs. We selected IFN-γ since previous studies showed that IFN-γ controlled the expression of chemokines such as Cxcl-9, Cxcl-10, and Cxcl-11 from the PT epithelial cells [39,40]. Additionally, the link between kidney injury markers such as KIM-1 and IL-18 and IFN-γ had been documented [54,55]. Moreover, a higher level of IFN-γ was associated with HEV induced tissue damages such as fulminant hepatitis failure and pregnancy [56,57]. In the absence of stimuli (HEV infection), we did not find a significant difference in the baseline level of IFN-γ released in the presence or absence of PBMCs cocultured with the PT cells. HEV infection did not increase the expression and release of IFN-γ from the PT cells in the absence of PBMCs. While a significantly higher level of IFN-γ was released in the presence of coculture of PBMCs with HEV-infected PT cells. IL-18 is a proinflammatory cytokine (known as IFN-γ inducing factor), increased the levels of circulating inflammatory cytokines such as TNF-α and IFN-γ in the presence of stimuli and resulted in a worsening of the acute kidney injury, and further damage to renal function [54]. Importantly, the treatment of PT cells alone with IFN-γ led to the upregulation of the chemokines without effect on the kidney injury markers. We hypothesized that the renal damage recorded during the course of HEV infection is a complex process that mediates by the interplay between HEV, renal epithelium, immune cells, and the signals produced during the infection process. Previously, we showed that HEV could infect the monocytes and macrophages and stimulate an inflammatory response from these cells [33]. Herein, we could characterize a possible signaling interaction between PT cells and PBMCs during the course of HEV infection which explains the mechanism of HEV mediated renal disorders. However, we could not exclude the possibility of other signaling pathways that could also lead to HEV directed renal damage.

**Figure 6:**
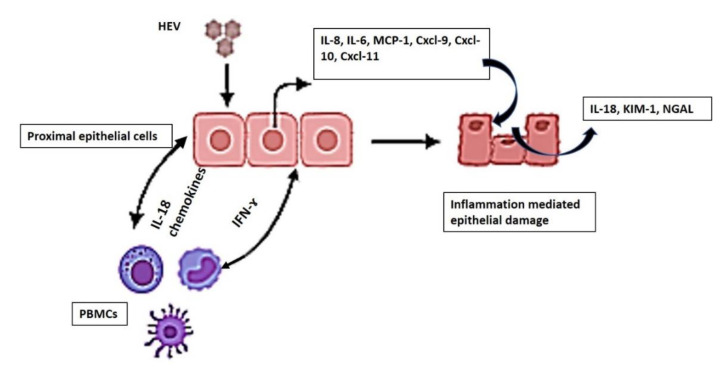
The crosstalk between the renal epithelium and PBMCs following HEV infection. Schematic showing the crosstalk between the epithelium and the immune cells (PBMCs) and the interplay between IFN-γ, chemokines, and IL-18 are the main mediators of HEV-induced renal epithelium damage.

Collectively, our results suggested that the crosstalk between PBMCs and PT epithelial cells during HEV infection and the signals axes IFN-γ/chemokines and IFN-γ/IL-18 and KIM-1 could be the mechanisms mediated by HEV during renal disorders (Figure 6). Likewise, high levels of IL-18 and IFN-γ were detected in chronically inflamed tissue in chronic granulomatous disease [41].

## 5. Conclusions

We developed a human primary PT culture system for HEV replication. HEV alone is not nephrotoxic and could not induce high inflammatory and chemokine responses. The crosstalk between immune cells and epithelium and consequently the signal axes IFN-γ/chemokines and IFN-γ/IL-18 and KIM-1 could be the immune-mediated mechanisms driven by HEV during the renal disorders.

## Supplementary Materials

The following are available online at https://www.mdpi.com/2076-393X/8/3/454/s1, Figure S1: Infection of the CD10^+^/CD13^+^ PT epithelial cells and PBMCs with UV-inactivated HEV inoculum. Infection of polarized CD10^+^/CD13 PT cells (A) and/or PBMCs (B) with UV-inactivated HEV-1 inoculum. Intracellular (dotted line) and extracellular (solid line) HEV RNA was quantified by qPCR. LOQ: limit of quantification. Depicted are the mean values of three independent experiments ± SEM. Figure S2: Effect of UV inactivated HEV-1 on the coculture of PBMCs with the PT epithelial cells CD10^+^/CD13^+^ PT cells were challenged with UV-inactivated HEV-1 for 7 days and then PBMCs from the same donors were added for an additional 3 days. Total cellular RNA was extracted from the PT cells and the mRNA expression level of proinflammatory markers (IL-8, IL-6, MCP-1, TNF-α, and IL-1β) (A), chemokines (Cxcl-9, Cxcl-10, and Cxcl-11) (B), and kidney injury transcripts (KIM-1, NGAL, and IL-18) (C) were assessed. The relative gene expression was determined by comparing the expression levels of these transcripts with mock cells (PT + PBMCs). Black columns represent unchallenged cells, and grey columns represent UV inactivated HEV challenged cells, Table S1: The sequences of the primers used in the study.
