# Supplementary material for: Hepatitis E Virus Mediates Renal Injury via the Interaction between the Immune Cells and Renal Epithelium
Source: Vaccines (Basel). 2020 Aug 14;8(3):454. doi: 10.3390/vaccines8030454 (PMC7564770; doi:10.3390/vaccines8030454)
Supplement: Supplementary file 1 [file vaccines-08-00454-s001.pdf]

Supplementary Materials:

Table S1. The sequences of the primers used in the study.

Gene	Primer sequence 5′-3′	Product Size (Bp)
18srRNA	Forward GTAACCCGTTGAACCCCATT<br>Reverse CCATCCAATCGGTAGTAGCG	151
MUC-1	Forward GTGCCCCCTAGCAGTACCG<br>Reverse GACGTGCCCCTACAAGTTGG	123
E-cadherin	Forward ATTTTTCCCTCGACACCCGAT<br>Reverse TCCCAGGCGTAGACCAAGA	109
Aquaporin 1	Forward CAACTTCAGCAACCACTGGATTT<br>Reverse GACCCCTTCTATTTGGGCTTCA	207
ICAM-1	Forward AGCTTCGTGTCCTGTATGGC<br>Reverse TTTTCTGGCCACGTCCAGTT	70
IL-6	Forward TGAACTCCTTCTCCACAAGCG<br>Reverse TCTGAAGAGGTGAGTGGCTGTC	151
IL-8	Forward ATGACTTCCAAGCTGGCCGTGGCT<br>Reverse TCTCAGCCCTCTTCAAAAACTTCTC	292
IL- β1	Forward AGCCATGGCAGAAGTACCTG<br>Reverse CCTGGAAGGAGCACTTCATCT	116
MCP-1 (CCL2)	Forward AGTCTCTGCCGCCCTTCT<br>Reverse GTGACTGGGGCATTGATTG	93
TNF-α	Forward CCCCAGGGACCTCTCTCTAA<br>Reverse CTCAGCTTGAGGGTTTGCTAC	109
Cxcl-9	Forward AGTGCAAGGAACCCCAGTAG<br>Reverse AGGGCTTGGGGCAAATTGTT	112
Cxcl-10	Forward: CCACGTGTTGAGATCATTGCT<br>Reverse: TGCATCGATTTTGCTCCCCT	152
Cxcl-11	Forward: GAGTGTGAAGGGCATGGCTA<br>Reverse: ACATGGGGAAGCCTTGAACA	71
IFN-α	Forward: CCTGATGAATGCGGACTCCA<br>Reverse: TAGCAGGGGTGAGAGTCTTTG	265
IFN-β	Forward: CGCCGCATTGACCATCTA.<br>Reverse: GACATTAGCCAGGAGGTTCTC.	112
KIM-1	Forward: CTG CAG GGA GCA ATA AGG AG<br>Reverse: ACC CAA AAG AGC AAG AAG CA	213
NGAL	Forward: GGGAAGTGGTATGTGGTAGG<br>Reverse: AGGGAAGACGATGTGGTTT	423
IL-18	Forward: GATAGCCAGCCTAGAGGTATGG<br>Reverse: CCTTGATGTTATCAGGAGGATTCA	121

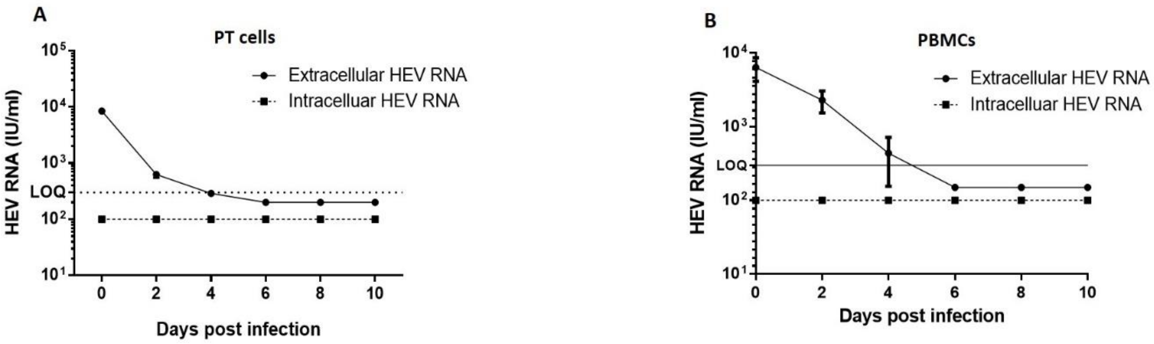

Figure S1. Infection of the $CD10^+$/$CD13^+$ PT epithelial cells and PBMCs with UV-inactivated HEV inoculum. Infection of polarized $CD10^+$/$CD13^+$PT cells (A) and/or PBMCs (B) with UV-inactivated HEV-1 inoculum. Intracellular (dotted line) and extracellular (solid line) HEV RNA was quantified by qPCR. LOQ: limit of quantification. Depicted are the mean values of three independent experiments ± SEM.

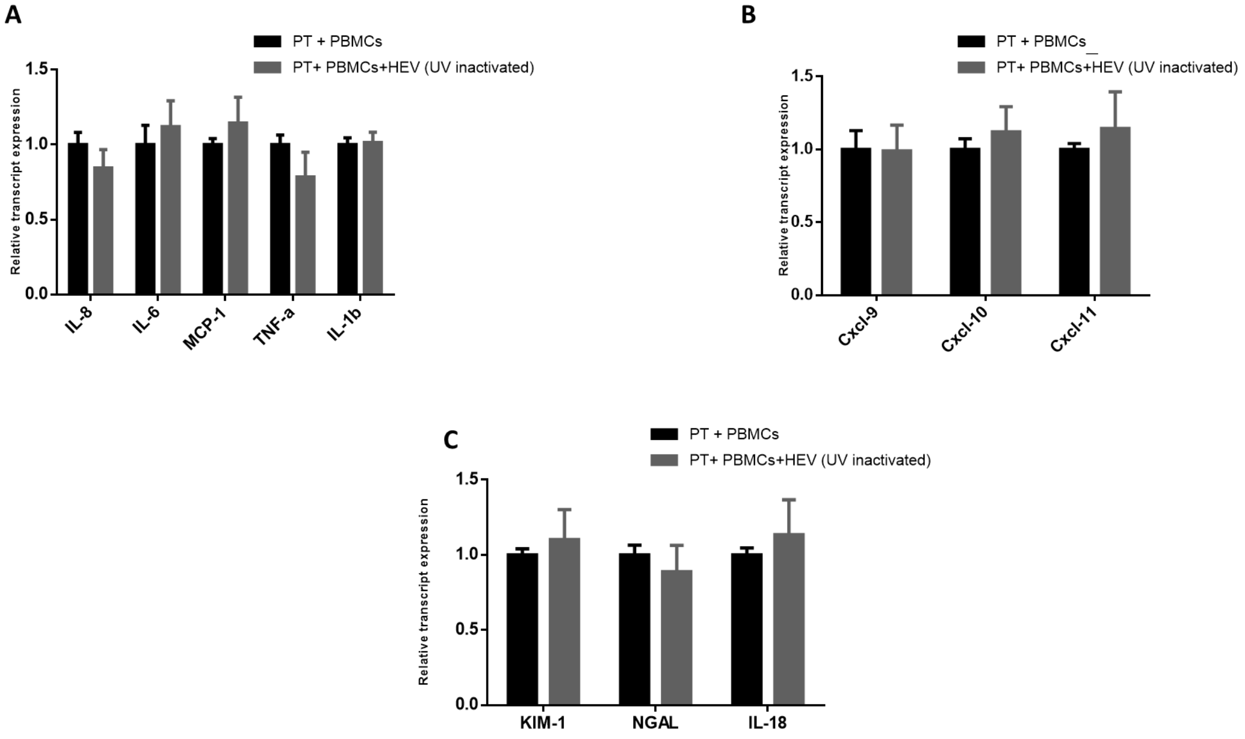

Figure S2. Effect of UV inactivated HEV-1 on the coculture of PBMCs with the PT epithelial cells $CD10^+$/$CD13^+$ PT cells were challenged with UV-inactivated HEV-1 for 7 days and then PBMCs from the same donors were added for an additional 3 days. Total cellular RNA was extracted from the PT cells and the mRNA expression level of proinflammatory markers (IL-8, IL-6, MCP-1, TNF-α, and IL-1β) (A), chemokines (Cxcl-9, Cxcl-10, and Cxcl-11) (B), and kidney injury transcripts (KIM-1, NGAL, and IL-18) (C) were assessed. The relative gene expression was determined by comparing the expression levels of these transcripts with mock cells (PT +PBMCs). Black columns represent unchallenged cells, and grey columns represent UV inactivated HEV challenged cells.

© 2020 by the authors. Submitted for possible open access publication under the terms and conditions of the Creative Commons Attribution (CC BY) license (http://creativecommons.org/licenses/by/4.0/).
